# Acoustic Emission Mechanisms During Polymer Processing and Chain Orientation: From Amorphous to Crystalline

**DOI:** 10.3390/polym17212948

**Published:** 2025-11-05

**Authors:** Guowei Chen, Tizazu Mekonnen

**Affiliations:** 1 National Engineering Research Center of Biomaterials, Nanjing Forestry University, Nanjing 210037, China; 2 Department of Chemical Engineering, Waterloo Institute for Nanotechnology, Institute of Polymer Research, University of Waterloo, Waterloo, ON N2L 3G1, Canada; tmekonnen@uwaterloo.ca

**Keywords:** acoustic emission, polymer crystallization, polymer processing

## Abstract

Acoustic emission (AE) technology has emerged as a highly sensitive and non-destructive method for the real-time monitoring of defect formation and microstructural changes during the manufacturing and early service life of polymeric materials and composites. This review highlights the fundamental principles and applications of AE in detecting crystallization-induced defects, such as cavities, dislocations, and microcracks, as well as plastic deformation mechanisms, including chain orientation, cavitation, and stress release. It is shown that AE activity correlates strongly with crystallinity and processing conditions, providing critical insights into microstructure–property relationships. The possible mechanisms can be the friction between grain boundaries, the local stress release, chain movement, phase changing, and fiber/filler debonding, among others. A comprehensive understanding can help with the prediction/prevention of early defects in the crystalline polymer processing. Furthermore, integrating AE with artificial intelligence and multi-sensor data fusion offers promising pathways toward smart, adaptive manufacturing systems capable of real-time quality control and early defect diagnosis in high-performance polymer composites.

## 1. Introduction

Owing to their excellent and editable properties, polymer and its composite materials are widely used in various critical fields, such as electronics, construction, automobile, aerospace, and renewable energy production [1,2,3]. However, defects are prone to occur during manufacturing, and the deformation of polymer products while in-service can significantly compromise structural safety and service life. Therefore, effective non-destructive testing (NDT) methods for accurate damage identification, localization, and evolution monitoring are of great engineering and scientific significance [4,5].

In recent years, multiple NDT techniques have been successfully applied to the quality evaluation and damage diagnosis of polymer composites, including ultrasonic testing (UT), acoustic emission (AE), terahertz pulsed spectroscopy (TPS), infrared thermography (IR), and X-ray computed tomography (CT), among others [6]. These methods have demonstrated their high value and good performance in detecting defects, such as debonding, delamination, and voids, in fiber-reinforced polymer (FRP) composites [7,8,9,10,11,12]. Nevertheless, each technique has its own advantages and limitations, which often falls short of providing a quick and cost-effective diagnosis of the early defects, especially during the manufacturing process and early-service life. Particularly in advanced carbon fiber-reinforced polymers (CFRP) and additively manufactured fiber-reinforced thermoplastic composites (FRTPCs), manufacturing defects (e.g., porosity, interlayer fusion flaws, fiber misalignment) and their propagation under fatigue and loading conditions impose higher requirements on NDT technologies [10,11,13]. In the case of CFRP, CT stands out in terms of detection accuracy, damage localization, and interface characterization [4]. For FRTPCs produced via additive manufacturing, micro-computed tomography (μ-CT) and structural health monitoring (SHM) systems integrated with self-sensing fibers significantly enhance the detection capability of micron-scale defects [13]. UT and TPS are also used to demonstrate the real defects and internal non-impregnated voids inside the polymer composite materials during the machining and manufacturing process [9,11]. However, these methods lack mobility, flexibility, and cost performance. Most importantly, the above methods cannot achieve dynamic monitoring and analysis on the formation and evolution process of the generated defects during the manufacturing, machining and early-service stage.

Acoustic emission (AE) is one of the most promising and cost-effective NDT methods for early defect detection within polymer composites. Owing to its passive and non-invasive characteristics, this method can be deployed during manufacturing and service operation, providing real-time data related to the initiation and progression of many kinds of defects. It operates by detecting and analyzing the elastic waves generated by “irreversible” phenomena—including crack initiation and growth, friction, debonding, and delamination, among others. These signals are captured by transducers and converted into electrical waveforms, which convey source-specific information regarding both the location and identification of certain mechanical behaviors [14,15,16]. In addition, it is believed that the real-time damage fracture behaviors and their development can be monitored and analyzed online via AE in a continuous mode [17,18,19].

During the manufacturing, machining, and the early-service period, the polymer and polymer composites experience great changes in temperature and stress fields. There might be further crystallinity and plastic deformation-induced phase transmission (e.g., from disordered to ordered structure), accompanied by stress release and chain movement. Cavities, cracks, and voids may come along and form defects within the polymer and its composites when it is changing from amorphous to crystalline [20,21]. This review critically evaluates and summarizes recent progress in the research regarding the employment of AE mechanisms during polymer processing and plastic deformation in the following sections: (1) Section 2 reviews AE applied for polymer and polymer composites; (2) Section 3 reviews polymer crystallization and the in situ study; (3) Section 4 reviews the AE mechanisms of polymers from amorphous to crystalline, and (4) Section 5 discusses future possibilities of AE for early defect inspection of crystalline polymer composites. This paper offers a concise review of these areas and provides an outlook of the potential directions for future research.

## 2. AE Applied for Polymer and Polymer Composites

### 2.1. Basic AE Theories and Parameters

Acoustic emission (AE) is recognized as an NDT method that captures material activity through emitted elastic waves. Recent advances have spurred the growing interest in adapting AE for continuous SHM applications. The technique detects high-frequency acoustic signals, typically between 10 kHz and 1 MHz, though lower ranges are also accessible, depending on the setup and hardware property. The AE stress or elastic waves are generated by internal damage behaviors, such as delamination or crack growth under stress [22]. The exceptional sensitivity of AE is underscored by its ability to detect signals with energies in the order of attojoules (10^−18^ J). This enables the identification of micro-cracking initiation and other subtle damage mechanisms beyond the reach of conventional techniques. For perspective, the kinetic energy of a common mosquito (e.g., 2.5 mg in weight, flying at 10 cm/s) is approximately 1.25 × 10^−8^ J, ten orders of magnitude larger than the detection threshold of AE [14].

**Figure 1:**
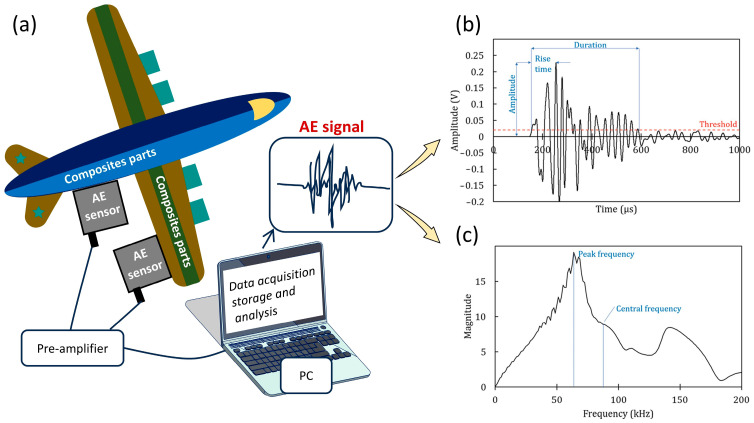
(**a**) Common units of AE system; (**b**) a typical AE signal in time domain; (**c**) AE signal in frequency domain. Figures (**b**,**c**) are adapted from [14] with permission.

A typical AE system includes sensors (transducers), pre-amplifiers, data acquisition/digitalization/storage/analysis units, and a PC, as shown in Figure 1a. In most cases, the AE data acquisition and analysis units are integrated with a personal computer (PC). The sensors are typically mounted on the surface of the test material, with a viscous coupling agent, such as petroleum jelly or bearing grease, applied at the interface to ensure efficient acoustic transmission. The sensors convert the surface mechanical pressure into electrical signals, which are then pre-amplified and digitized for acquisition. In addition to full waveform recording, which is commonly supported by modern signal acquisition systems, key signal parameters are also extracted and stored for analysis (Figure 1b,c, adapted from [14]). The parameters show the featured characteristics of certain signals, which helps explain the AE sources. The commonly used parameters employed in AE analysis are defined and listed in Table 1 [22,23]:

### 2.2. AE as NDT for Polymer and Polymer Composites

The Kaiser effect in AE has been observed during the stretching of pure polymers, such as acrylonitrile-styrene (ANS) and acrylonitrile butadiene styrene (ABS) at deformations exceeding 1% [24]. It was observed that, in the glassy state, crazes or microcracks generated during stretching remain as permanent defects; whereas, in the rubbery state, such damage is self-healing. This distinction is reflected in the AE response, which is closely associated with void formation in polymers. The stress cracking behavior of polybutylene terephthalate (PBT) in ethanol was investigated using tensile and stress relaxation tests. Meanwhile, AE was employed during mechanical testing to detect and characterize internal failure mechanisms [25]. AE proved to be highly effective in elucidating the mechanical behavior and distinguishing between different testing conditions.

In the context of polymer and its composite materials, AE serves as an effective NDT and SHM technique for identifying and tracking damage mechanisms, including fiber breakage, matrix cracking, delamination, and debonding, either under mechanical loading or during service [17,26,27]. AE was also applied for bio-based polymer composites and biological materials [18,28,29,30]. Thus, it is an effective method to demonstrate the mechanical behaviors of polymer and polymer composites.

The AE technique leverages piezoelectric sensors, such as piezoelectric ceramics and polymers, which can be embedded within laminated FRP composites to form “smart materials”. They are capable of continuous condition monitoring, offering an integrated alternative to conventional external NDT methods [22]. In addition, AE activity has been observed to initiate as early as 10% of ultimate failure stress, and intensifies with increasing load [31]. Nevertheless, integrating AE sensors into composite structures involves challenges related to sensor size, placement, and interfacial coupling, which can affect measurement accuracy and reliability [32]. In addition, the AE data can be combined with artificial intelligence (AI) to further increase the processing efficiency. Studies, such as that by Guo et al. [33], have demonstrated the applicability of deep learning models in classifying AE data related to different damage modes.

Furthermore, the integration of AE with other techniques can enhance both data processing efficiency and accuracy. For instance, AE combined with a microscope was employed to show the hierarchical mechanical behaviors of natural bamboo in a previous study. Acoustic signals can be confirmed by visualized images [34,35]. AE was also used with digital image correlation (DIC), and the potential of data fusion was examined for detecting, identifying, and estimating the remaining life in structural polymer composites [36]. In another work, AE provided early damage monitoring in the fatigue testing of polymers, including nanochannels that offer self-healing ability. Fatigue testing was combined with dynamic mechanical analysis (DMA). Its primary objective is to halt the test based on acoustic wave features that act as a precursor indicator of a fracture process zone, allowing for termination prior to the detection of stiffness degradation [37]. AE is a promising NDT and SHM method with increasing possibilities and functionalities for the structure monitoring of polymer and polymer composites.

## 3. Polymer Crystallization and the In Situ Study

### 3.1. Crystalline Polymer Classifications and Applications

Currently, more than 50% of the most consumed thermoplastics globally are crystalline or semicrystalline polymers [38]. Crystalline (including semicrystalline) polymers are a class of polymers whose molecular chains can form dominant crystalline regions developed from the amorphous state. Common types of crystalline polymers include general purpose and engineering categories [39,40,41]. In high-performance composites, crystalline polymers are used as matrices reinforced by engineering fibers (e.g., carbon fiber, glass fiber), significantly enhancing the overall mechanical properties and functionalities. For instance, polyetheretherketone (PEEK) and polyphenylene sulfide (PPS) combined with carbon fiber are often used to manufacture aircraft interior components, door parts, and wing structures, among other components [42]. Their high specific strength and high-temperature resistance meet the demands for weight reduction and reliability in aviation [40,42]. Liquid crystal polymers (LCP) and polyamide (PA)-based composites are used in precision connectors, engine components, and heat-resistant housings, leveraging their excellent dimensional stability and creep resistance [43,44,45]. Additionally, PEEK and polyimide (PI) composites are used in many biomedical applications, including medical implants and imaging device components, due to their biocompatibility and radiation resistance [3,46]. Moreover, crystalline polymer-based composites are also employed in insulation, robotic structural parts, and precision transmission systems [2,47,48]. Through fiber and filler reinforcement, crystalline polymer-based composites achieve outstanding properties, such as high strength, high modulus, corrosion resistance, biocompatible, and fatigue resistance, making them essential materials in high-end manufacturing and applications.

### 3.2. Crystallization Mechanisms

**Figure 2:**
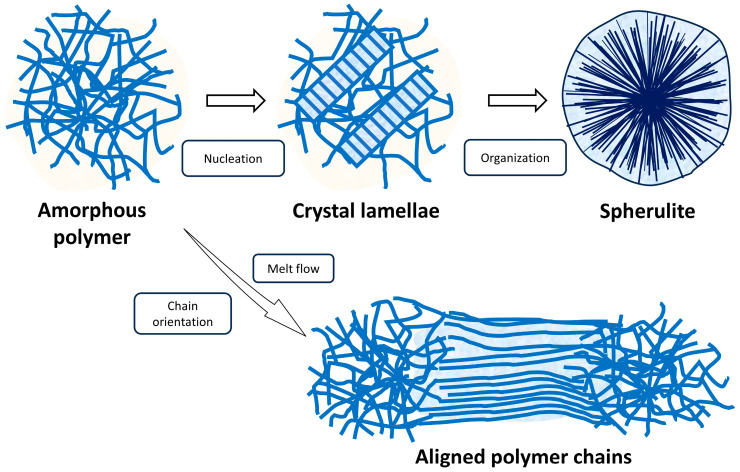
The polymer crystallization process and mechanisms through nucleation, melt flow, and chain orientation.

Polymer crystallization fundamentally involves the rearrangement of polymer chains through segmental motion into ordered crystalline regions. Their ability to crystallize depends on factors such as functional group polarity, chain regularity, flexibility, and processing conditions [38,49]. The crystallization mechanism of polymers is a kinetic process governed by molecular structure, thermodynamic conditions, and external fields [50]. Polymers with regular chain structures (e.g., polyethylene (PE)) first form nuclei—via homogeneous or heterogeneous nucleation—during cooling from the melt. These nuclei then grow into lamellae, which further organize into higher order structures, such as spherulites [51]. Orientation-induced polymer crystallization was studied by Nitta [52]. In the polymer system, the intermolecular interaction between polymer chains based on quantum mechanics was introduced. During melt flowing or under tensile orientation, the proximity between the protons of adjacent extended chains induces an attractive force, governed by interactions among hydrogen atoms surrounding the main chains. This interaction results in a splitting of energy into ground and excited states. The spontaneous transition to the ground state drives orientation-induced crystallization. The mentioned crystallization process and mechanisms are displayed in Figure 2.

Crystallinity, crystal form, and morphology are strongly influenced by cooling rate, temperature profile, molecular weight, and nucleating agents. In most cases, slower cooling rates and higher crystallization temperatures generally promote more perfect crystals, while external fields (e.g., flow or stretching) significantly enhance oriented crystallization, thereby improving the mechanical properties and thermal stability of the material [38]. During flow-induced crystallization, the pressure and shear flow rate are the two main factors that can alter the dynamic conditions of polymer crystals for both nucleation and growth process. However, the mechanisms are still missing, which demands further experimental and simulating works [53].

Recent advances in understanding key yet underexplored factors governing the crystalline morphology of crystallizable polymers were reviewed by Kay Saalwächter et al. [54]. Particular emphasis was placed on the role of intracrystalline chain dynamics and the impact of entanglements within the amorphous regions. Computational simulations were used to offer novel insights into the thermodynamic mechanisms driving the structural organization of these materials. Polymer crystallization under flow or large deformation were also examined by using computer modeling methods in a study by Yamamoto [55]. It was believed that, in polymer systems subjected to elongational or shear flow, molecular chains, particularly longer ones, become stretched, leading to the formation of central fibrillar structures. These subsequently serve as nucleation sites for the growth of chain-folded lamellar crystals and higher-ordered structures.

**Figure 3:**
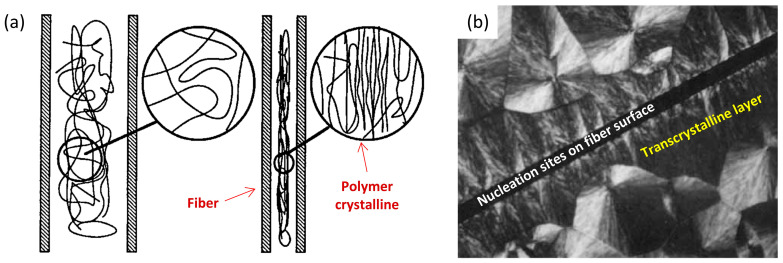
The effects of fibers on the crystallization of polymer molecules: (**a**) free amorphous molecular chains and localized crystalline aligned the fibers; (**b**) nucleation sites and the induced transcrystalline layer around the fiber. Adapted from [56] with permission.

In the case of crystalline polymer composites, the fiber or fillers have a great influence on the polymer crystallinity. The incorporation of reinforced fibers and/or fillers enhances the crystallization kinetics by accelerating the crystallization rate, elevating the degree of crystallinity, and refining crystal size, particularly at low filler loadings [42]. These effects can be attributed to three main mechanisms: (1) the alignment of polymer molecules along the fillers or fibers, facilitating localized crystalline ordering; (2) the provision of nucleation sites on the filler surfaces; and (3) the formation of a transcrystalline layer induced by high nucleation density, which contributes to reduced crystal dimensions [56]. The mechanisms are illustrated in Figure 3. There might be defects or imperfection when the fiber or fillers are introduced. Thus, a thorough understanding of the crystallization process is crucial for the manufacturing of polymer composites, especially for high-end applications.

### 3.3. Defects or Imperfection Induced by Crystallization

During polymer crystallization, defects and imperfections arise due to the inherent complexity of macromolecular packing and kinetic constraints. Common defects include point defects (vacancies, interstitial atoms), dislocations, cavities, and impurities [20,55,56,57,58]. The underlying causes can be summarized as follows: (1) the long chain nature of polymers prevents perfect alignment, entanglements and varying chain lengths lead to kinetic trapping during crystallization [57]; (2) rapid crystallization rates, commonly seen in industrial processing, limit the molecular mobility, reducing the time available for chains to reach equilibrium positions and resulting in metastable structures with abundant imperfections [59]; (3) the presence of chemical inhomogeneities (e.g., copolymers, catalysts residues) or physical impurities disrupts regular packing [60,61].

**Figure 4:**
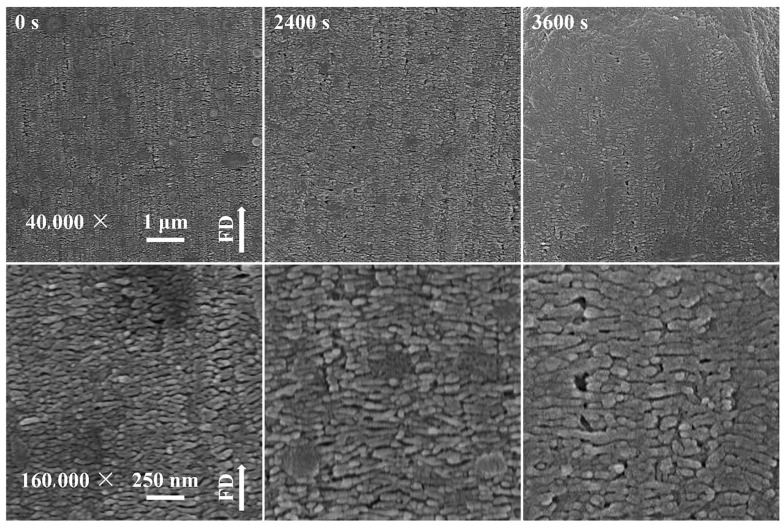
Polymer (iPP) crystallization under controlled flow rate and pressure. Voids and dislocations are observed at 3600 s. Reprinted from [62] with permission.

**Figure 5:**
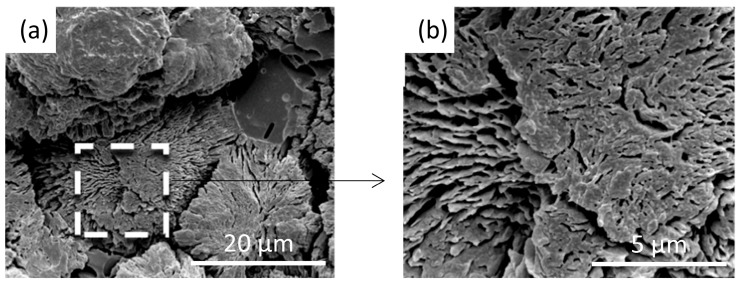
PLA/PEG blends crystalline morphology under controlled flow rate and pressure. (**a**) The voids and grain boundary cracks within PLA spherulites; (**b**) enlarged image of the voids. Adapted from [64] with permission.

The crystallization process of isotactic polypropylene (iPP) was studied under controlled flow rate and pressure [62], and the morphology was visualized, as shown in Figure 4. It was indicated that the iPP lamellae grew thicker over time; however, visible voids and dislocations were also observed when thicker lamellae were generated (3600 s). Unfortunately, the authors did not mention the generating mechanisms of these voids and dislocations. Theoretically, this might be due to the stress release during crystallization [63]. The crystallization of polylactic acid (PLA) blended with polyethylene glycol (PEG) under flow and pressure fields was investigated [64]. The voids within PLA spherulites and clear grain boundary cracks were clearly observed, as displayed in Figure 5. This probably resulted from the inhomogeneity and stress release-induced cracks along grain boundaries during the crystallization process.

These defects and imperfections during crystallization may significantly impact material properties by adding cracks and stress concentration sites within the polymer and polymer composites. Therefore, for the forming, manufacturing, and machining process of polymer and polymer composites, it is essential to proactively identify crystallization-induced defects (e.g., voids, cracks) using rapid and efficient testing methods, such as AE.

### 3.4. In Situ Characterizations of Polymer Crystallization During Processing

Observations on the polymer crystallization process offer fundamental insights into the kinetics and mechanisms of macromolecular development under realistic manufacturing conditions. The in situ characterization of polymer crystallization is powerful to advance the understanding of the macromolecule structure and phase changing, and to enable the precise control of material performance, providing critical guidance for optimizing product properties. This necessitates ultra-high time resolution techniques capable of capturing the real-time structural evolution under complex thermomechanical fields.

Several in situ characterization methods have been integrated within polymer processing. Their advantages and disadvantages are listed in Table 2. Ultrasound technology, sensitive to variations in acoustic properties such as velocity and attenuation, has been used to monitor crystallization during injection molding [65]. For instance, velocity changes in linear low-density polyethylene (LLDPE) correlate with mold cavity pressure drop and subsequent crystallization. Optical methods, based on changes in turbidity and birefringence, have been applied to track crystallization kinetics in various polymers, though limitations exist for low-crystallinity materials like PET [53,66]. A previous study demonstrated that terahertz pulsed spectroscopy (TPS) serves as a unique tool for the non-destructive monitoring of polymer composite manufacturing. TPS effectively tracks binder polymerization via picosecond dynamics and detects internal voids using time-of-flight tomography, highlighting its potential for online process control [11]. Synchrotron-based X-ray scattering (SAXS/WAXS) provides segment-level and long-range structural details and has been successfully applied to processes like film blowing and 3D printing [67,68]. However, its application in injection molding remains challenging due to the confined mold cavity, high pressure, and rapid timescales involved. Moreover, X-ray characterization is considerably more expensive than acoustic and optical techniques. It is therefore crucial to develop strategies that balance cost and operational efficiency.

## 4. Acoustic Emission Mechanisms of Polymers from Amorphous to Crystalline

AE has been successfully used to monitor the crystallization of inorganics and metals [71,72,73]; however, limited research has been conducted on the AE mechanisms of polymers during crystallization and processing. Furthermore, as mentioned in Section 2.2, AE has been widely used during mechanical testing, but the initial plastic deformation stage in polymers and polymer composites has often been overlooked due to the scarcity of detectable AE signals. Nonetheless, these early signals may hold critical information regarding chain re-orientation and friction and phase transformation mechanisms. Therefore, this section will focus specifically on elucidating the AE mechanisms associated with polymer crystallization, encompassing both processing conditions and the initial stages of deformation.

### 4.1. AE Mechanisms During Crystallization

**Figure 6:**
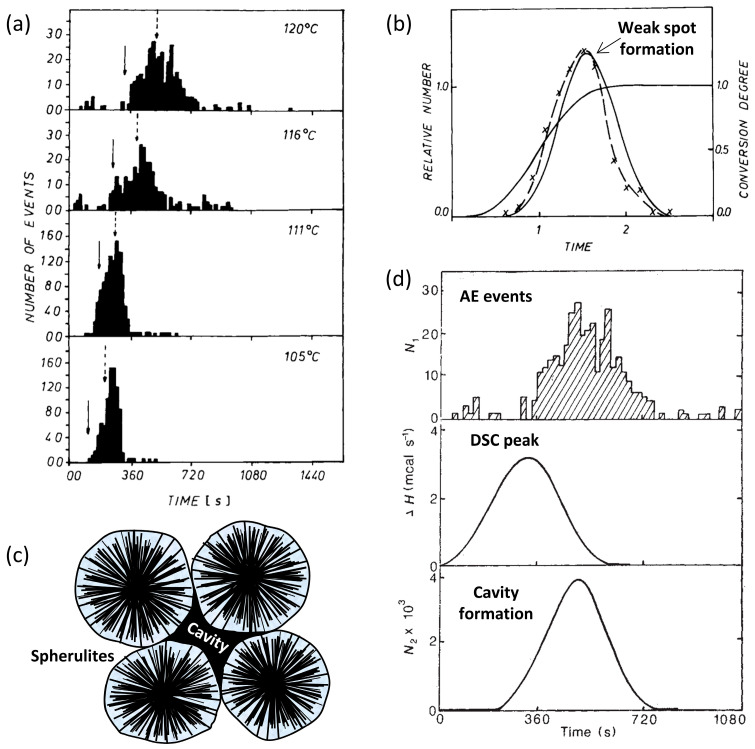
AE mechanisms during the crystallization of iPP: (**a**) AE events over varied temperatures, where the solid and dashed arrows correspond to the half-time of crystallization and the peak formation rate of quadruple boundary points under the assumption of athermal nucleation, respectively; (**b**) weak spot formation during the crystallization; (**c**) the schematics of spherulites and the cavity between boundaries; (**d**) the correlation of AE events, heat of fusion generated during isothermal crystallization (DSC peak), and formation of cavity. Figures (**a**,**b**,**d**) are modified from [74] with permission.

As has been discussed in Section 3.3, defects such as dislocations, cavities, and im-perfections can happen during the crystallization process. Galeski et al. detected AE during the isothermal crystallization of polymers such as poly(methylene oxide) (POM), high-density polyethylene (HDPE), and isotactic polypropylene (iPP) [74,75]. The generated AE signals were recorded during the crystallization of iPP at different temperatures, as shown in Figure 6a. Decreasing the temperature caused the shift of AE signals at an earlier stage, shortening the whole crystallization period. In addition, the AE signals were much stronger at lower temperatures, which is probably due to the larger scale of stress release. The authors proposed that AE originates from the abrupt release of stress accumulated within these cavities, caused by the density changes during crystallization. The formation of a weak spot (quadruple boundary points) in terms of mathematical statistics was described [74], as shown in Figure 6b. Significant AE activity was observed only in iPP and POM, wherein the microstructure revealed the presence of cavities between spherulites [76], as displayed in Figure 6c. Thus, the formation of quadruple boundary points can be confirmed by the evidenced cavities. By contrast, HDPE and iPP with 1 wt.% of talc exhibited negligible AE signals that formed only small spherulites with limited cavities. A comparison with DSC data indicated that the majority of AE signals occurred toward the end of crystallization, with the onset of AE coinciding with the exothermic peak in the DSC curve, as shown in Figure 6d. The correlation was not explicitly discussed but evident from the figures. In other words, the crystallization and the formation of cavities can be correlated with the recorded AE signals.

The AE mechanisms during heat-cooling cycles were reported in non-isothermal crystallization studies of PP [24,77]. Significant groups of AE events were triggered with a sudden temperature jump from room temperature to 100 °C. When the specimen was withdrawn, cooled, and re-immersed in boiling water, AE activity reoccurred but was markedly weaker than during the first jump. It is unfortunate that the origin of AE sources accompanying rapid thermal cycles remains unclear and requires further investigation.

Some of the studies referenced above were published over 40 years ago and, unfortunately, attracted limited follow-up research over the subsequent decades. However, with the growing emphasis on high-performance polymer composites today, quality control during manufacturing has become increasingly critical. A key factor is the polymer–fiber/filler interface, which can be significantly influenced by the crystallization behavior of the polymer matrix. Crystallization-induced cavities or substantial stress release may lead to interfacial debonding, severely compromising the mechanical properties of crystalline polymer composites.

### 4.2. AE Mechanisms of Chain Orientation During Initial Plastic Deformation

Previous studies have shown that there are correlations between AE signals and polymer molecule events [78,79]. Nevertheless, numerous questions and challenges remain. For instance, the AE sources generated during polymer chain orientation require clearer classification. AE is sensitive to the cracks within bulk polymer materials [80]. However, distinguishing whether AE signals originate from polymer chain mobility rather than micro-crazing or cracking remains a significant challenge during the plastic deformation of crystalline polymers.

**Figure 7:**
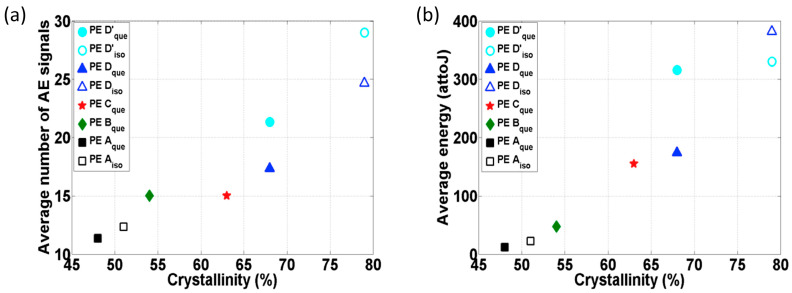
AE signal number and average energy over the crystallinity during the initial plastic deformation of PE: (**a**) average AE signal numbers over the crystallinity; (**b**) average AE signal energy over the crystallinity. Adapted from [81] with permission.

The initiation of plastic deformation of PE during tensile tests have been investigated [81]. It was indicated that the AE activities (signal numbers and average energy) increased significantly with the crystallinity, as shown in Figure 7. The formation of cavitation was associated with a substantial release of energy, which significantly enhanced both the acoustic activity and the energy of the AE signals. However, the respective contributions of chain movement and cavitation to AE generation have not yet been decoupled.

**Figure 8:**
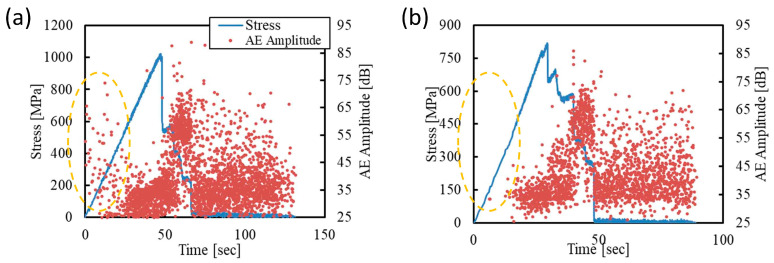
AE signals marked in yellow circles were collected during the initial plastic deformation of PA: (**a**) 0 h heat treatment; (**b**) 1 h heat treatment. Adapted from [83] with permission.

Polyamides (PA) exhibit strong intermolecular hydrogen bonding between amine hydrogen and carbonyl oxygen atoms, which stabilizes the fully extended all-trans conformation of the chains within the crystalline regions. These robust interactions significantly restrict slip between crystalline stems, distinguishing their mechanical behavior from that of polymers relying solely on dispersion forces (e.g., PE) where molecular slip occurs more readily [82]. PA composites subjected to varying durations of thermal treatment were tested under static bending, with AE used to monitor the deformation process [83]. The results indicated that heat treatment increased crystallinity and significantly reduced AE activity during plastic deformation, as shown in Figure 8. Although not explicitly discussed in the study, the detected signals may originate from chain mobility or re-orientation within the amorphous regions, or the interface friction between PA crystals.

**Figure 9:**
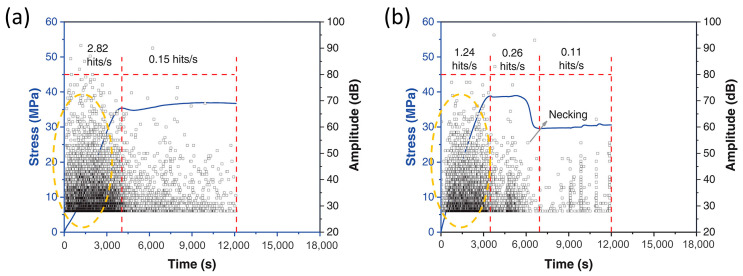
AE signals marked in yellow circles were collected during the initial plastic deformation of PBT: (**a**) no ethanol; (**b**) covered by ethanol. Adapted from [25] with permission.

In a similar study, the molding temperature was used to control the crystallinity of polybutylene terephthalate (PBT), and ethanol was used to stimulate the environmental fluid [25]. The results implied that PBT covered by ethanol showed much less intensive AE signals in the initial deformation process, as displayed in Figure 9. This effect may result from the plasticizing influence of ethanol, and consequently the locally plasticized regions exhibited reduced sensitivity to stress concentration. For the specimen with higher crystallinity, the plasticizing effect was less obvious. This is probably due to the regular molecular structure that provided more nucleation sites for cracks [84].

During the manufacturing and machining of polymers and polymer composites, structural evolution occurs at the molecular level through chain re-orientation during both crystallization and plastic deformation. These microstructural changes are critical to the final material properties, yet they are often difficult to monitor in real time without destructive testing. AE technology offers promising non-destructive means to detect and analyze these underlying mechanisms.

## 5. Future Possibilities of AE for Early Defect Inspection of Crystalline Polymer Composites

AE technology holds significant potential for early defect inspection in crystalline polymer composites during processing and manufacturing. Its high sensitivity to micro-scale structural changes, such as crystallization-induced stress release and cavity formation, which enables the real-time detection of incipient defects long before they become critical. By analyzing AE features, including energy, frequency, duration, and amplitude, it is possible to distinguish between different failure modes and to identify their origins from the environmental noise, whether from imperfect crystallization, thermal stress, uneven flow, or mechanical overloading during the initial deformation.

Integrating AE sensors into production equipment, such as 3D printing, extruders, injection molds, compression molds, and autoclaves, enables the real-time, in-process monitoring of crystalline polymer composites during critical manufacturing stages [85,86]. These sensors detect the elastic waves generated by microstructural events, including crystallization onset, crystal–crystal friction, cavitation, dislocation, and cavity formation. In processes like injection molding, where cooling rate and shear flow directly affect crystallinity and morphology, AE provides immediate feedback on whether crystallization is proceeding uniformly/mildly or with defects. By analyzing key AE parameters, such as energy, amplitude, and frequency, the system can identify deviations (e.g., imperfect crystallization, premature solidification, or inhomogeneous crystal growth) and trigger self-adjustment. The outcome is not only higher quality composites but enhanced process efficiency and reduced energy consumption.

There are significant challenges in enhancing the accuracy and flexibility of AE. While AE can theoretically pinpoint the location, including depth, of active defects in polymer composites, its effectiveness diminishes when defects are confined to a very small area or specimen. In such cases, even minor deviations in acoustic wave propagation are magnified, leading to substantial positioning errors. Furthermore, AE is inherently less capable than imaging techniques like ultrasound or X-ray in characterizing defect size. This limitation is particularly critical during processes like polymer crystallization, where defects can be microscopic or even nanoscale. Consequently, integrating AE with other non-destructive testing (NDT) methods presents a promising strategy for obtaining a comprehensive understanding of defect characteristics.

With further development in multi-sensor (such as acoustic, temperature, pressure, and optical) data fusion [87,88,89] and artificial intelligence (AI) assisted systems [90,91,92], this complex non-destructive method could become a cornerstone of smart manufacturing systems for next-generation polymer composites. The automatic adjustments to temperature, pressure, or dwelling time, enhances reliability, prolongs service life, and enables more sustainable production through waste reduction and energy-efficient processing. This closed-loop adaptive control significantly reduces scrap rates, improves repeatability, and ensures that final components meet strict structural and functional requirements.

## 6. Conclusions

This review has examined the fundamental mechanisms and practical applications of acoustic emission (AE) technology in monitoring polymer crystallization and early-stage deformation processes. AE demonstrates high sensitivity to microstructural changes, including void formation, stress release, chain rearrangement, and crystal–crystal interactions, making it a powerful tool for the non-destructive, real-time detection of defects during manufacturing and initial service phases. The analysis of AE features, such as energy, frequency, duration, and amplitude, enables differentiation between failure modes and links signal characteristics. Despite its potential, several challenges remain. The individual contributions of chain mobility and cavitation to AE signals have not been fully decoupled, particularly during the early plastic deformation of the crystalline polymers. Moreover, practical limitations such as sensor integration under high temperature and pressure, signal interference in noisy industrial environments, and the need for advanced signal processing techniques must be addressed to maximize AE’s utility. Looking forward, future efforts should focus on the quantitative correlations between AE data and microstructural evolution, supporting the development of smart manufacturing systems with enhanced reliability and performance for next-generation polymer composites.

## Figures and Tables

**Table 1:** AE parameters and their definitions.

AE Parameters	Definitions
Event	The signal captured either in the frequency domain or the time domain, resulting from the generation of elastic waves. It represents the complete AE waveform acquired during testing
Hit	An AE signal that exceeds the user-defined threshold on a single channel. Multiple hits may originate from multiple events or channels
Amplitude	The peak amplitude of an AE hit, expressed in decibels (dB) or volts
Rise time	The time interval from the initial threshold crossing to the point of maximum amplitude
Counts	The number of pulses within an AE hit that surpass the predefined threshold
Duration	The time from the first threshold crossing to the last
Energy	The integral of the signal envelope over the duration of the hit
Peak frequency	The frequency component with the highest magnitude in the AE signal spectrum
Central frequency	The centroid or center of gravity of the AE frequency spectrum

**Table 2:** The advantages and disadvantages of some in situ characterization methods during polymer processing.

Method	Advantage	Disadvantage	Ref.
Ultrasound	Direct and efficient: sensitive to acoustic waves’ velocity and attenuation	Have to continuously emit ultrasonic waves, and timing is difficult to grasp	[65,69]
Optical method	Fast and imageable: depends on the light intensity and scattering pattern	Not suitable for low-crystalline polymers	[53,66]
THz spectroscopy	Quick imaging: capture changes of picosecond dynamics in media	Sensitive to temperature and moisture	[11,70]
X-ray imaging	High-throughput imaging and high resolution	Expensive; hard to catch the crystallization window; not through metal mold	[67,68]

## Data Availability

No new data were created or analyzed in this study. Data sharing is not applicable to this article.
